# The impact of shift work and organisational climate on nurse health: a cross-sectional study

**DOI:** 10.1186/s12913-018-3402-5

**Published:** 2018-07-27

**Authors:** Tessa Dehring, Kathryn von Treuer, Bernice Redley

**Affiliations:** 1 Faculty of Health, School of Psychology, Deakin University, Melbourne Burwood Campus, 221 Burwood Highway, Burwood, VIC 3125 Australia; 2 Faculty of Health, School of Medicine, Deakin University, Melbourne Burwood Campus, 221 Burwood Highway, Burwood, VIC 3125 Australia; 3 Cairnmillar Institute, 391-393 Tooronga Road, Hawthorn East, VIC 3123 Australia; 4 Centre for Quality and Patient Safety Research- Monash Health Partnership, School of Nursing and Midwifery, Faculty of Health, Geelong Campus, Deakin University, 1 Gheringhap Street, Geelong, VIC 3220 Australia; 5 Nous Group, Level 19, 567 Collins Street, Melbourne, VIC 3000 Australia

**Keywords:** Nurse health, Organisational climate, Shift work, Supervisor support, Task orientation

## Abstract

**Background:**

The negative effects of shift work schedules, specifically night and rotating shifts, have been widely reported. However, little is understood whether particular aspects of the organisational environment, related to specific shifts, may influence the negative impact of shift work. This study investigated the variation in organisational climate and health outcomes across shift work schedules (day, night, rotating).

**Methods:**

This cross-sectional study involved nursing staff (*n* = 108) who were all registered nurses from two Melbourne health services. There were slightly more nursing staff that participated from one health service *(n = 56)* than the other health service (*n = 52)*. Nursing staff completed a survey on either paper form or online which comprised of: demographic characteristics, organisational climate (work environment scale) and health outcomes (general health questionnaire).

**Results:**

The study found that organisational climate factors and health outcomes differed across shift types. Rotating shift staff exhibited significantly higher coworker cohesion scores when compared to night staff. Night staff reported significantly greater levels of physical comfort within their work environment than rotating staff. Overall, supervisor support emerged as a significant predictor of health outcomes such as somatic complaints, social dysfunction and overall distress. Task orientation was also shown to significantly predict levels of social dysfunction.

**Conclusions:**

Findings suggest that interventions with a focus on enhancing the organisational climate, focused in increasing supervisor support, may mitigate the potential negative health outcomes experienced by shift workers.

**Trial registration:**

Not applicable to this study.

## Background

Shift work-scheduled rosters are common in various industries in Australia [1]. Rosters such as rotating and night shifts have been linked to negative psychological and physical outcomes [2, 3]. These include increased sick days [2, 4]; non-prescription medication use [2]; cardiovascular disease [5, 6]; gastrointestinal complaints [2, 7]; and job-related stress [7, 8]. Shiftwork negatively effects job performance [3, 9]; general health [9]; and results in more frequent work-related injury and errors [4, 10].

Organisational climate describes the work environment and is comprised of factors such as social support (supervisor and co-worker), leader relations, role clarity, physical factors, job pressure and innovation [11, 12]. Organisational climate factors have also been shown to directly impact employees’ psychological and physical health [11]. Various studies have demonstrated a link between organisational climate and individual variables such as job satisfaction [13, 14], burnout [15], work performance [16], and job-related stress [14]. All of these factors can directly or indirectly affect employee health and wellbeing [11]. Little is known about differences in organisational climates between shifts and its impact on nurse health. Variation in organisational climates across shifts may affect nurses’ health. Since shiftwork is an inescapable part of nursing, organisational climate may provide an avenue to improve nurse health.

The relationship between organisational climate and nurses’ health was previously explored in Australia [12]. Several organisational climate factors predicted employee health across shift types. In comparison with day staff, rotating staff were more likely to demonstrate social dysfunction, and night staff had elevated depression scores. Work pressure was the most consistent predictor of employee health (anxiety, depression, social dysfunction, and somatic complaints). Increased co-worker cohesion was found to predict lower levels of social dysfunction across shift types.

The aim of the present study was to investigate the impact of shift work schedules and organisational climate on nurse health and determine whether previous findings by Von Treuer et al. [12] were generalisable. Three hypotheses were outlined. Hypothesis 1 (H1) Rotating and night staff would report higher rates of acute distress (i.e. anxiety, depression, social dysfunction, and somatic complaints) than day staff. Hypothesis 2 (H2) Organisational climate would differ for nurses across the different shift types. Hypothesis 3 (H3) Organisational climate factors would predict health outcomes in shift workers, whereby work pressures, control, involvement, and social support might contribute most to nurse health outcomes.

## Methods

### Participants

The sample consisted of 108 participants comprised of 98 females and 10 males with a mean age of 40 years (*SD* = 12.19). There were slightly more nursing staff that participated from one health service *(n = 56)* than the other health service (*n = 52)*. Participants worked fixed day shift (*n* = 37), fixed night shift (*n* = 13) and rotating day and evening shift (*n* = 58). Most of the participants had been working their shift type for 3 years or more (*n* = 88, 82%) and had been with their organisation for over 3 years (*n* = 76, 70%). The majority of participants worked in critical care (44%) or surgical (20%) areas and were full time (42%) or part time (58%) employees. The participants were front-line employees (67%), supervisors (22%), managers (9%) or casual staff (2%). Approximately 1500 nurses received the invitation to participate in the study (approximately 7% response rate). However, the actual response rate is hard to determine, due to email inbox technical difficulties and unknown numbers of staff who are on leave or absent for other reasons.

### Procedure

Two metropolitan healthcare centres (Site 1, Site 2) agreed to participate and data was collected during 2013. Low risk ethical approval and consent was obtained from both health services; Eastern Health Research and Ethics Committee (LR74/1213); Epworth HealthCare Human Research Ethics Committee (LR114–13) and the Deakin University Human Research Ethics Committee (2013–113). Consent from all three committees was received in written form. Participants were instructed through the Plain Language Statement and the online survey that completion of the survey documented their consent to participate. This form of participant written consent was approved by the ethics committees. Participation was anonymous. Participants were invited to complete the online questionnaire in two ways, either via an email invitation distributed by an independent third party in the organisation or through an advertisement in the organisation’s newsletter. One of the healthcare centres requested a combined approach utilising online and hard copy methods. Therefore 224 hard copy questionnaires with reply-paid envelopes were distributed to the nurse managers on each of the wards.

### Materials

#### Organisational climate

The Work Environment Scale – Real (WES-R) [17] consists of 90 statements requiring a “yes” or “no” response, which measure 10 organisational climate factors: involvement, co-worker cohesion, supervisor support, autonomy, task orientation, work pressure, clarity, control, innovation and physical comfort. The WES-R subscales have demonstrated adequate validity and reliability [18]. In this study, the reliability estimates for the autonomy (.44) and control (.47) subscales were inadequate (< 0.6) and therefore omitted. Cronbach’s alpha ranged from 0.62 to 0.74 for the remaining climate factors, similar to previous research [12, 19]. The reliability estimates were as follows: involvement (.69), co-worker cohesion (.63), supervisor support (.74), task orientation (.65), work pressure (.62), clarity (.68), innovation (.73) and physical comfort (.72).

#### Health

The General Health Questionnaire (GHQ-28) [20, 21] assessed the general wellbeing of participants. The GHQ-28 consists of 28 items that are positively (*n* = 7) or negatively (*n* = 21) worded. The GHQ-28 produced a total score representing overall psychopathology, and four subscale totals (somatic complaints, anxiety and insomnia, social dysfunction and depression). A total score of above 13 was considered to be “acutely distressed” [22]. Cronbach’s alpha-estimated reliability of the GHQ ranged from 0.80 to 0.92 for the present study, consistent with prior research [12, 23].

The studied applied several statistical analyses which included logistic regression, MANOVA and Roy–Bargmann stepdown analysis.

## Results

### Data screening

Preliminary data screening revealed missing values distributed randomly across the items and participants; Little’s MCAR χ^2^ = 1922.161, *p* > 0.05. Expectation maximisation was used to replace missing values. Scale totals were constructed from item level responses and were subsequently screened for violations of normality and evidence of outliers. All variables exhibited acceptable levels of skew and kurtosis [24]. Univariate outliers (> ±3.29 standard deviations from the mean) were identified for the social dysfunction subscales of the GHQ. These scores were within the possible range of scores and had negligible effects on parameter estimates when comparing analyses with and without the cases; therefore, the decision was made to retain these cases without transformation. An examination of Mahalanobis distance revealed no multivariate outliers (*p* < 0.001).

### Health outcomes by shift type

The results show that shift types differed in their mean scores on each of the health outcomes and organisational climate (see Table 1). On average, day shift staff exhibited a lower score on two of the five indices of health: social dysfunction and depression. In comparison, rotating shift workers exhibited the highest levels of psychological distress (as indicated by the GHQ total score), levels of anxiety and somatic symptoms. Moreover, night shift workers exhibited the highest scores relating to social dysfunction. However, the different shift types (rotating, night and day) revealed no significant differences for health outcomes subscales (*p* > 0.05). The within-group variability on each of the health indices were similar for each of the shift types.

**Table 1:** Mean scores (SD) for health outcome and organisational climate by shift type

Variable	Day (*n* = 37)	Night (*n* = 13)	Rotating (*n* = 58)
Health Outcomes			
Somatic Complaints	4.00 (2.32)	3.08 (2.40)	4.36 (2.08)
Anxiety and Insomnia	4.19 (2.32)	3.92 (2.47)	4.74 (2.43)
Social Dysfunction	1.81 (1.81)	2.00 (1.41)	1.88 (1.53)
Depression	1.11 (2.18)	1.62 (2.57)	1.50 (2.18)
GHQ Total	11.11 (6.53)	10.62 (7.56)	12.48 (6.48)
Organisational climate			
Job Involvement	48.91 (9.80)	47.85 (10.70)	49.88 (8.42)
Coworker Cohesion	54.43 (9.10)	50.77 (12.63)	57.60 (8.79)
Supervisor Support	50.41 (10.67)	44.46 (13.70)	47.69 (10.59)
Task Orientation	53.22 (10.47)	55.23 (8.96)	56.86 (8.32)
Job Pressure	58.43 (8.71)	59.69 (10.06)	59.81 (6.74)
Role Clarity	45.95 (12.03)	47.07 (9.25)	48.03 (9.05)
Innovation	51.08 (11.16)	47.54 (8.32)	49.26 (10.65)
Physical Comfort	47.54 (11.22)	50.08 (12.20)	43.55 (9.51)

Thirty eight percent of day and night shift staff were classified as “acutely distressed”. In comparison, 47% of rotating shift respondents were classified as “acutely distressed”. Logistic regression using distress status (acute vs. not acute) as the dependent variable (DV) and dichotomised shift work variables (day vs. night, day vs. rotating) as independent variables (IV) failed to demonstrate significant odds ratios (*B* = 0.143, *p* > 0.05, odds ratio = 2.32).

### Organisational climate profile across shift types

Organisational climate factors varied across shift type (see Table 1). Rotating staff exhibited the most positive perceptions of their work environment, reporting the highest levels of job involvement, co-worker cohesion, task orientation, and role clarity (Table 1). The within-group variability in levels of role clarity was shown to differ significantly among shift types (*F*(2,105) = 4.22, *p* < 0.05).

MANOVA and Roy–Bargmann stepdown analysis examined whether organisational climates differed across shift types. Given that the current study is a replication of a previous study [12] a similar order of priority was allocated to the DVs. The climate factors that were assigned the highest priority included coworker cohesion, job involvement, task orientation and innovation. These factors were followed by supervisor support, work pressure, role clarity and physical comfort, as studies have shown inconsistencies in whether they exhibit cross shift differences [12]. The results from this analysis are provided in Table 2.

**Table 2:** The univariate effects exhibited by each organisational climate factor

Dependent variable	Stepdown *F*	df1	df2	*η* ^*2*^	95% CI around *η*^*2*^	
					Lower	Upper
Coworker Cohesion	3.31*	2	105	.06	.00	.15
Job Involvement	0.76	2	104	.01	.00	.07
Task Orientation	1.64	2	103	.03	.00	.11
Innovation	1.58	2	102	.03	.00	.11
Supervisor Support	1.98	2	101	.04	.00	.12
Work Pressure	0.02	2	100	.00	.00	.01
Role Clarity	0.22	2	99	.00	.00	.04
Physical Comfort	3.22*	2	98	.06	.00	.16

*Note*. *p = .041 **p = .047; CI = confidence interval

The multivariate effect for shift type was significant (Roy’s largest root = 0.20, *F*(8, 99) = 2.52, *p* = 0.02). Perceived level of cohesion was found to vary by shift type (*F*(2, 105) = 3.31, *p* = 0.041, *η* ^2^ = 0.06). Post hoc comparisons revealed that this univariate effect was attributable to differences between night (*M* = 50.77) and rotating (*M* = 57.60) shift staff. Levels of physical comfort differed significantly according to shift type (*F*(2, 96) = 3.16, *p* = 0.047, *η* ^2^ = 0.06). The night staff (*M* = 50.08) reported higher levels of physical comfort than did rotating staff (*M* = 43.55).

### Organisational climate and health outcomes

Individual regressions were conducted to evaluate the relative contributions of organisational climate factors for predicting the overall and four indices of psychopathology-somatic complaints, anxiety and insomnia, depression, social dysfunction (Table 3).

**Table 3:** Regression results for health indices

Dependent Variable	Significant IVs	*R* ^2^	Adj *R*^2^	Δ*R*^2^	*B*	*SE*	*β*	*sr*
Somatic Complaints	Supervisor Support	.11*	.04	.11*	−.06*	.03	−.28	−.21
Anxiety and Insomnia	–	.09	.01	.09	–	–	–	–
Social Dysfunction	Task Orientation	.18**	.12	.18**	−.04**	.02	−.24	−.19
	Supervisor Support	.18**	.12	.18**	−.04**	.02	−.25	−.18
Depression	–	.16**	.09	.16**	–	–	–	–
GHQ Total	Supervisor Support	.17***	.10	.17***	−.16***	.08	−.27	−.19

Note: *p = 0.03, **p = 0.047, ***p = 0.04

Overall, organisational climate factors made a significant contribution to the prediction of social dysfunction (Δ*R*^2^ = 0.18, *p* = 0.008), depression (Δ*R*^2^ = 0.17, *p* = 0.018), and GHQ total score (Δ*R*^2^ = 0.17, *p* = 0.009). Supervisor support made a significant unique contribution in predicting somatic complaints (*B* = − 0.06, *p* = 0.03), social dysfunction (*B* = − 0.04, *p* = 0.047), and GHQ total score (*B* = − 0.16, *p* = 0.04). The degree of task orientation was a significant unique predictor of social dysfunction (*B* = − 0.04, *p* = 0.047). None of the individual organisational climate factors predicted differences in levels of depression or anxiety and insomnia.

## Discussion

The current study compared the organisational climate and health outcomes of nurses across different shift types.

### Shift-related differences in nurse health outcomes

The results revealed that scores on each of the health indices varied across shift types. Contrary to H1, the proportion of night and day staff classified as “acutely distressed” was the same. There were no statistical differences between shift groups for GHQ scores. This finding may be due in part to inadequate power from the small sample size of night staff. A high proportion of individuals were classified as “acutely distressed” in each of the shift types and these rates appear increased from previous study estimates [12]. In alignment with previous research [12] it was hypothesised that rotating shift workers would experience greater levels of social dysfunction than day and night staff. This was not supported by our results and the inconsistencies in the relationship between shift work and social dysfunction warrants further research.

### Shift-related differences in Organisational climate

H2 was partially supported. Only co-worker cohesion was significantly higher for rotating staff compared to night staff, consistent with previous studies [12]. The perception of reduced co-worker cohesion exhibited by night staff may be due to the reduced number of staff working overnight, which may result in less opportunity to interact with colleagues. Fixed night staff are likely to have less opportunity to interact with colleagues on other shift types—such as the day shift—outside of a handover process. In contrast, rotating staff would have the opportunity to work with a range of colleagues on both fixed day and night shifts, contributing to a higher level of co-worker cohesion.

Level of physical comfort emerged as an organisational climate factor that also differed between shift types. Night staff reported significantly higher levels of physical comfort than rotating staff. The physical environment experienced by rotating staff is highly variable, which may hinder feelings of comfort. Another possible explanation could be that night staff have less exposure to conditions that cause physical discomfort (i.e. heat and noise) as these may be less prevalent at night. Scores on the other organisational climate factors were generally consistent across the three shift types. Scores on organisational climate factors emerged as quite similar across the three shift types. This is in contrast with our hypothesis that the organisational climate of night shift would differ most when compared to rotating and day staff.

### Influence of Organisational climate on health outcomes

H3 was only partially supported by the results. The combined effects of organisational climate factors on health outcomes were non-trivial, explaining between 9 and 18% of the variance in health. Supervisor support was the single organisational climate factor that emerged as a significant contributor to the prediction of health outcomes of nurses. Specifically, supervisor support was shown to significantly predict level of somatic complaints, social dysfunction, and overall psychological distress. The protective role of supervisor support in buffering employees from the potential negative effects of shift work has been reported in several studies [25–27]. The results suggest that supervisor support could mitigate negative health outcomes, such as somatic complaints including migraines, fever, and generally feeling run down. The finding aligns with previous studies [28], where social support (both co-worker and supervisor) was found to be preventative for somatic symptoms such as headaches and gastric problems. What remains unclear is how supervisor support has the ability to prevent somatic symptoms in employees. The potential role of stress as a mediator or moderator of this relationship could be explored in future studies.

Social dysfunction was minimised by supervisor support. One indicator of supervisor support was whether participants felt they were able to be open with their supervisor about issues such as asking for a pay rise and discussing personal problems. It may be that the openness of the supervisor-employee relationship influences social functioning. Task orientation emerged as an additional organisational climate factor that significantly predicted lower social dysfunction. One explanation is that the more focused nurses are at work, the more efficient they are in completing the tasks as part of their role, and the less impact there will be on their social functioning. They are likely to be able to leave on time and have less work-to-family spillover. In contrast to previous findings by Von Treuer et al. [12], work pressure, involvement, and co-worker cohesion did not significantly predict any of the health indices.

As the results of this study vary from those of von Treuer et al. [12] which suggests that the results from the earlier study cannot be considered generalisable, though findings may also reflect changes over time. Nevertheless, the findings of this study highlight the importance of an effective supervisor-employee relationship and uncovered significant gaps in current understanding about the impact of shift work on employee and organisational outcomes.

### Limitations

The current study was a correlational study which prevents the ability to draw causal inferences about the relationship between organisational climate and health outcomes. The major limitation was small sample size, subsequent low power, and a potential type two error. Nurses are renowned for being an over surveyed population and this may also provide some insight into our response rate due to overexposure or ‘survey fatigue’. Furthermore, specific situational stressors may also impact findings. For example, one of the healthcare centres involved in the study was undergoing organisational accreditation during the recruitment phase of the study. Although some previous findings were replicated, a claim of generalization to Australian nurses more broadly would need to be tested.

### Practical implications

The study found that rotating staff were experiencing the most psychological distress. Interestingly, night and day shift staff were found to be experiencing similar rates of psychological distress, which suggests that a fixed style roster is more protective for nurse health. Alternatively, organisations might reduce the rate of rotations for those staff on a rotating shift to help to minimise the potential for negative outcomes. Shift work organisations might focus on techniques to enhance supervisor support. The finding that levels of co-worker cohesion were highest for rotating staff suggests that ensuring that employees have the opportunity to engage with others outside of their teams and business units during the shift may help buffer the impact of shift work. These findings need further investigation as they suggest that modifiable aspects of the organisational climate can be used to minimise the negative impacts of shift work on health.

## Conclusion

The present study demonstrated that shift types differ in terms of their organisational climate and the level of psychopathology-somatic complaints, anxiety and insomnia, depression, social dysfunction experienced by individuals. The present findings are generally aligned with prior studies, the general consensus being that nursing is a demanding and stressful profession, often associated with psychological distress. The findings suggest that supervisor support and task orientation may have a protective role in employee health. Interventions targeted at organisational climate may assist in mitigating the potential negative impacts of shift work schedules on employees’ health.

## Abbreviations

DV: Dependent variable
GHQ-28: The general health questionnaire
H1: Hypothesis 1
H2: Hypothesis 2
H3: Hypothesis 3
IV: Independent variables
WES-R: Work environment scale – real
